# Longitudinal Survey of Fungi in the Human Gut: ITS Profiling, Phenotyping, and Colonization

**DOI:** 10.3389/fmicb.2019.01575

**Published:** 2019-07-10

**Authors:** Stefano Raimondi, Alberto Amaretti, Caterina Gozzoli, Marta Simone, Lucia Righini, Francesco Candeliere, Paola Brun, Andrea Ardizzoni, Bruna Colombari, Simona Paulone, Ignazio Castagliuolo, Duccio Cavalieri, Elisabetta Blasi, Maddalena Rossi, Samuele Peppoloni

**Affiliations:** ^1^Department of Life Sciences, University of Modena and Reggio Emilia, Modena, Italy; ^2^Department of Molecular Medicine, University of Padua, Padua, Italy; ^3^Department of Surgery, Medicine, Dentistry and Morphological Sciences with Interest in Transplant, Oncology and Regenerative Medicine, University of Modena and Reggio Emilia, Modena, Italy; ^4^Department of Biology, University of Florence, Firenze, Italy

**Keywords:** gut microbiota, fungi, colonization, metagenomics, *Candida*

## Abstract

The fungal component of the intestinal microbiota of eight healthy subjects was studied over 12 months using metagenome survey and culture-based approaches. *Aspergillus*, *Candida*, *Debaryomyces*, *Malassezia*, *Penicillium*, *Pichia*, and *Saccharomyces* were the most recurrent and/or dominant fungal genera, according to metagenomic analysis. The biodiversity of fungal communities was lower and characterized by greater unevenness, when compared to bacterial microbiome. The dissimilarities both among subjects and over the time within the same subject suggested that most of the fungi passed through the gastro-intestinal tract (GIT) without becoming stable colonizers. Certain genera, such as *Aspergillus* and *Penicillium*, were isolated in a minority of cases, although they recurred abundantly and frequently in the metagenomics survey, likely being environmental or food-borne fungi that do not inhabit the GIT. *Candida* genus was recurrently detected. *Candida albicans* isolates dominated among the cultivable mycobiota and longitudinally persisted, likely as commensals inhabiting the intestine or regularly reaching it from *Candida*-colonized districts, such as the oral cavity. Other putative colonizers belonged to *Candida zeylanoides*, *Geotrichum candidum*, and *Rhodotorula mucilaginosa*, with persisting biotypes being identified. Phenotyping of fungal isolates indicated that *C. albicans* adhered to human epithelial cells more efficiently and produced greater amounts of biofilm *in vitro* than non-*albicans Candida* (NAC) and non-*Candida* fungi (NCF). The *C. albicans* isolates also induced the highest release of HBD-2 by human epithelial cells, further differing from NAC and NCF. Nine representative isolates were administered to mice to evaluate the ability to colonize the intestine. Only two out of three *C. albicans* strains persisted in stools of animals 2 weeks after the end of the oral administration, whereas NAC and NCF did not. These results confirm the allochthonous nature of most the intestinal fungi, while *C. albicans* appears to be commonly involved in stable colonization. A combination of specific genetic features in the microbe and in the host likely allow colonization from fungi normally present solely as passengers. It remains to be established if other species identified as potential colonizers, in addition to *Candida*, are true inhabitants of the GIT or rather reach the intestine spreading from other body districts.

## Introduction

The complex microbial community hosted in the gastrointestinal tract (GIT) of humans and animals is composed of bacteria, archaea, fungi, protozoa, and viruses (Qin et al., 2010; Huseyin et al., 2017). The complexity of the GI bacterial population, accounting up to 10^12^ microorganisms per gram of content, has been widely investigated, while abundance, role, and diversity of fungi in the human gut are still understudied (Huseyin et al., 2017). Cultivable fungi in feces range between 10^2^ and 10^6^ cfu/g (Simon and Gorbach, 1984), representing a minor component of gut microbiota, and their genes cover less than 0.1% of the whole microbial metagenome (Qin et al., 2010). Although several fungal species are harbored in the normal GIT their potential role in host health status have been only partially investigated (Seed, 2014; Suhr and Hallen-Adams, 2015; Strati et al., 2016; Nash et al., 2017; Auchtung et al., 2018; Borges et al., 2018).

Most studies on the human mycobiome have been focused on opportunistic pathogens, such as *Candida* species, and in particular *Candida albicans* (Mayer et al., 2013; Merseguel et al., 2015; Cavalieri et al., 2018). This species colonizes oropharynx, genital, and gastrointestinal mucosa of 30–70% of healthy individuals, and colonization can evolve into infection under certain circumstances, such as decay of host immune defense or structural impairment of muco-cutaneous barriers (Brandt and Lockhart, 2012). Disruption of mucosal barriers and host immune defenses impairment may lead to endogenous candidiasis arising from commensal strains (Yan et al., 2013). Indeed, *C. albicans* is the most virulent species of the genus *Candida*, due to several virulence factors and fitness attributes that contribute to the pathogenic potential (Cavalieri et al., 2018). The first step in the infection onset is the interaction between *Candida* and host cells (Moyes et al., 2015). *C. albicans*, as well as other *Candida* spp., expresses several adhesins, which bind to extracellular proteins of epithelial cells. Adhesion of fungi to the mucosal cell membrane may stimulate biofilm formation, that increases the resistance to host immune defenses and antifungal drugs. As many opportunistic pathogens, *C. albicans* takes advantage from biofilm formation to escape host immune defenses and antifungal therapy as well (Tsui et al., 2016). Other *Candida* species, such as *C. dubliniensis*, *C. glabrata*, *C. guilliermondii*, *C. kefyr*, *C. krusei, C. tropicalis*, and *C. parapsilosis* can reside as commensal in diverse niches of the human body and may be responsible of biofilm-associated severe infections (Muadcheingka and Tantivitayakul, 2015). Currently, the significant increase of infections caused by other *Candida* spp. is matter of clinical concern, because of their resistance to conventional antifungal agents (Merseguel et al., 2015; Wu et al., 2017).

Nowadays, high throughput next generation sequencing (NGS) has become a common technology for microbiome studies and has overtaken traditional microbiological methods for the identification of members of the microbial communities. The knowledge on the bacterial component of the gut microbiota has received a major impulse from 16S rRNA gene profiling. Likewise, NGS targeting the Internal Transcribed Spacers (ITS) within eukaryotic rRNA genes allows to characterize the intestinal fungal population (Tang et al., 2015). Studies investigating the composition of gut mycobiota identified tens of fungal operational taxonomic units (OTUs), most of which ascribed to *Aspergillus*, *Candida*, *Cladosporium*, *Clavispora*, *Cyberlindnera*, *Debaryomyces*, *Galactomyces*, *Malassezia*, *Penicillium*, *Pichia*, *Rhodotorula*, and *Saccharomyces* (Hoffmann et al., 2013; Seed, 2014; Suhr and Hallen-Adams, 2015; Strati et al., 2016; Suhr et al., 2016; Nash et al., 2017; Auchtung et al., 2018; Borges et al., 2018). Recently, a strain specific analysis using whole genome sequencing of single isolates was applied to study strain specific features associated with the ability of *Saccharomyces cerevisiae* to colonize the human gut, showing that strains from different origin differ in the ability to adapt to the gut environment (Ramazzotti et al., 2019).

Several factors affect the description of the mycobiota by culture-independent sequencing, including non-standardized methods of DNA preparation, the target of primers for amplification, the consistency of taxonomic information in reference databases, and the characteristics of the cohort (e.g., geographical location, diet, and climate) (Nash et al., 2017). In particular, the selection of the best markers for profiling fungi is debated, since ITS1 has been regarded as the best target for profiling fungi, while the most recent studies indicate ITS2 as more accurate (Wang et al., 2015; Yang et al., 2018). However, the clustering and taxonomic capacities did not differ between ITS1 and ITS2, that generate similar patterns of community structure (Yang et al., 2018).

The ecological and functional role of intestinal fungi, the source (allochthones or autochtones), the interaction with the host, and the capability to permanently colonize or to transit through the GIT are topics that still deserve deep investigation (Huseyin et al., 2017). The aim of the present study was to investigate the presence and persistence of fungi in the feces of eight healthy subjects using a combination of culture-based approaches and metagenomics. The fungal isolates were phenotypically characterized, assessing their ability to colonize the murine GIT, adhere to human Caco-2 cells and induce the release of human β-defensin 2 (HBD2), and to form biofilm *in vitro*. In particular, the behavior of *C. albicans*, non-*albicans Candida* (NAC), and non-*Candida* fungi (NCF) was compared.

## Materials and Methods

### ITS and 16S Amplicon Libraries, Sequencing, and Analysis

DNA was extracted from feces using the FastDNA SPIN Kit for Feces (MP-Biomedicals, United States). DNA quality was checked on 1% agarose gel TAE 1X and quantified with a NanoDrop spectrophotometer. Fungal ITS1 rDNA region was amplified with a fusion primer set, consisting of 18SF and 5.8S1R primers (5′-GTAAAAGTCGTAACAAGGTTTC-3′ and 5′-GTTCAAAGAYTCGATGATTCAC-3′, Findley et al., 2013) coupled with adaptors, key sequence and barcode, according to 454 Sequencing System Guidelines for Amplicon Experimental Design (Roche, Switzerland). Bacterial V1-V3 hypervariable regions of the 16S rRNA gene were generated with universal 27-Forward and 533-Reverse primers (5′-AGAGTTTGATCMTGGCTCAG-3′ and 5′-TTACCGCGGCTGCTGGCAC-3′, Barelli et al., 2015) coupled with adaptors, key sequence and barcode (Roche, Switzerland). ITS1 and V1-V3 regions were amplified according to Strati et al. (2016) and Barelli et al. (2015), respectively. The PCR products were electrophoresed, cleaned using the AMPure XP beads kit (Beckman Coulter, United States), and quantified via quantitative PCR using 454 Kapa Library Quantification Kit (KAPA Biosystems, United States).

Equimolar amounts of purified amplicons were pooled into ITS1 and 16S amplicon libraries, that were subjected to pyrosequencing on the Roche GS FLX+ system using the XL+ chemistry (Roche, Switzerland). Pyrosequencing reads were processed using the MICCA pipeline for trimming and quality filtering (Albanese et al., 2015). Quality filtered sequences were processed with Qiime 2 pipeline (Bolyen et al., 2018) for closed-reference OTU picking, taxonomy assignation, and diversity metrics computation. For the fungal microbiota, the OTU picking and taxonomy assignation were carried out utilizing UNITE ver. 7.2 Dynamic Classifier^1^ as reference database with the similarity threshold set at 0.97. For the bacterial microbiota, the closed-reference OTU picking and taxonomy assignation were carried out utilizing as reference SILVA SSU database release 132^2^ with the similarity threshold set at 0.97.

ITS1 and 16S rRNA gene sequences have been submitted to NCBI repository with the BioProject ID: PRJNA545913.

### Culture-Dependent Enumeration of Fecal Yeast-Like Fungi

Fresh feces were collected from 8 healthy adult volunteers (hereinafter referred to as V1–V8), 4 males and 4 females aged 25–55, enrolled among the employees of the University of Modena and Reggio Emilia and their relatives. The subjects were not in relationship with the researchers, followed a western omnivore diet, and had not been treated with prebiotics and/or probiotics for 1 month and antibiotics for at least 3 months before being enrolled and throughout the study. Written informed consent was obtained from all the enrolled subjects, in accordance with the protocol approved by the local research ethics committee (reference number 225-15, Comitato Etico Provinciale, Azienda Policlinico di Modena, Italy).

Feces were collected and analyzed at the beginning of the study (sample I), 3 (sample II), and 12 months (sample III) later. Stool samples were collected in sterile containers and forwarded to the laboratory within 3 h. The fecal samples were serially diluted and 100 μL dispersed onto Potates Dextrose Agar (PDA, BD Difco, Franklin Lakes, NJ, United States) supplemented with 100 mg/L chloramphenicol and 100 mg/L ampicillin. The plates were aerobically incubated at 30°C for 72 h, then fungal colonies were counted and isolated.

### RAPD-PCR Analysis of Fungal Isolates and Taxonomic Attribution

For each fecal sample, 48 colonies from PDA plates were subjected to RAPD-PCR clustering. Genomic DNA was extracted and subjected to RAPD-PCR amplification according to Raimondi et al. (2017). The reaction was performed in 15 μL of a mixture consisting of Dream Taq Buffer (Thermo Fisher Scientific, Waltham, MA), 0.5 μM of M13-RAPD primer (5′-GAGGGTGGCGGTTCT-3′), 100 μM of each dNTP, 0.75 U Taq polymerase (DreamTaq, Thermo Fisher Scientific), and 50 ng of gDNA from the isolates. The thermocycle was the following: 94°C for 4 min; 45 cycles of 94°C for 1 min, 34°C for 1 min, and 72°C for 2 min; 72°C for 7 min. The PCR products were electrophoresed for 4 h at 160 V in a 25 × 25 cm 2% (w/v) agarose gel in TAE buffer. RAPD-PCR fingerprints were digitally captured, then analyzed with Gene Directory 2.0 software (Syngene, United Kingdom), which calculated similarities and derived a dendrogram with an unweighted pair group method with arithmetic means (UPGMA).

To attribute each biotype to a species, the ITS1 and ITS2 spacer regions were amplified, sequenced, and compared with GenBank database. Amplification was performed with ITS1 (5′-TCCGTAGGTGAACCTGCGG-3′) and ITS4 (5′-TCCTCCGCTTATTGATATGC-3′) primers within 150 μL of PCR Master Mix (Thermo Fisher Scientific), containing 0.2 μM of each primer, and 50 ng of gDNA. The thermocycle was the following: 95°C for 5 min; 30 cycles of 95°C for 1 min, 60°C for 1 min, and 72°C for 1 min; and 72°C for 7 min.

### Strain Typing of *C. albicans*

For microsatellite length polymorphism (MLP) typing, the loci CAI and CAIV were amplified by PCR with 5′ fluorescently labeled forward primer (6-carboxyfluorescein) according to Sampaio et al. (2005). The PCR products were injected in an ABI 370 genetic analyzer (Applied Biosystems, Foster City, CA, United States) and the fragment sizes were determined using the GeneScan 3.7 software. For single-strand conformation polymorphism (SSCP) electrophoresis analysis, approximately 100 ng of unlabeled PCR products were loaded on an acrylamide gel and separated with a DCode Universal Mutation Detection System (Bio-Rad, Hercules, CA, United States). Electrophoresis and silver staining were performed according to Li and Bai (2007).

### Biofilm Formation Assay

*Candida* isolates were grown overnight in SDA plates at 37°C. Yeast cells were washed with PBS and standardized to 1 × 10^5^ or 1 × 10^6^ yeast cells/mL in RPMI-1640 medium (Gibco, Grand Island, NY, United States) supplemented with 10% heat-inactivated Fetal Bovine Serum (hiFBS; Defined Hyclone, Logan, Utah, United States), 2 mM L-glutamine, and 50 mg/mL gentamycin, hereafter referred to as cRPMI. The yeast cell suspension was placed (0.1 mL/well) in a 96-well polystyrene microplate and incubated at 37°C for 24 and 48 h (Orsi et al., 2014). After incubation, the biofilm was quantified by the crystal violet (CV) assay. The cells were washed 3 times with PBS at room temperature, fixed with methanol, stained with 1% CV for 15 min, washed three times with distilled water, and treated with 33% acetic acid for 10 min. The color development, read at 540 nm (OD_540_), was proportional to biofilm. The consensus threshold for biofilm formation was established at OD_540_ = 1.5.

### Human Epithelial Cells

The human epithelial colorectal adenocarcinoma cell line Caco-2 was grown in 75-cm^2^ tissue-culture flasks (Nalge Nunc International, Naperville, IL, United States). When reaching 80–90% confluence, the cells were split 1:3 by standard methods. The cells were cultured at 37°C under 5% CO_2_ in Dulbecco’s Modified Eagle Medium (EuroClone, Milan, Italy) supplemented with 10% hiFBS, 2 mM L-glutamine, and 50 mg/mL gentamycin, hereafter referred to as cDMEM.

### Adhesion Assay

Caco-2 cells (6.5 × 10^5^/mL, 200 μL/well) were seeded in Lab-Tek II chamber slides, grown to confluence for 24 h at 37°C, then supplemented with a suspension of yeasts (2.6 × 10^6^/mL, 200 μL/well) in cRPMI at E:T ratio 1:2. The cells were incubated for 1.5 or 3 h at 37°C under 5% CO_2_. Fifteen minutes before the end of incubation, 40 μL/well of 1% Uvitex 2B (Polysciences Europe GmbH, Germany) were added. The wells were washed three times with PBS to remove the unbound yeast cells. The remaining yeasts were fixed for 30 min with PBS-buffered 4% paraformaldehyde. The Caco-2 cells were washed twice with PBS and then treated with PLGAR (Thermo Fisher Scientific, Waltham, MA, United States). The blue fluorescence of adhered yeasts was visualized by epifluorescence microscopy Nikon Eclipse 90i imaging system, equipped with Nomarski DIC optics (Nikon Instruments Inc., Melville, NY, United States). Samples were photographed with a DS-2Mv Nikon digital camera. The percentage of adhesion was defined as the ratio between Caco-2 cells with at least one adhering fungal cell and the total number of Caco-2 cells observed. At least 200 epithelial cells per sample were examined.

### Secretory Activity of Human Epithelial Cell Line Caco-2

Caco-2 cells suspended at 8.0 × 10^5^/mL in cDMEM, were seeded (200 μL/well) in 24 well-plates and allowed to reach confluence for 24 h at 37°C. Yeast cells (1.6 × 10^8^/mL, 1 mL/well, E:T ratio 1: 100), 500 μg/mL β-D-glucan, or 10 μg/mL LPS were added, and the plates were incubated for additional 24 h. The supernatants were collected and the level of HBD-2 was assessed by the enzymatic immunoassay β-2-Defensin Human (Phoenix Pharmaceuticals, Karlsruhe, Germany).

### Animal Trial

Nine intestinal yeast strains (*C. albicans* 02-10, *C. albicans* 04-10, *C. albicans* 08-06, *C. parapsilosis* 01-18, *Candida pararugosa* 04-14, *Candida zeylanoides* 01-03, *Geotrichum candidum* 02-01, *S. cerevisiae* 03-01, and *Rhodotorula mucilaginosa* 04-01) were grown for 24 h in 200 mL of Potato Dextrose Broth (BD Difco). The biomass was harvested by centrifugation, suspended in PBS containing 15% v/v glycerol to obtain single-dose aliquots of 10^7^ cells in 150 μL and stored at −80°C. Aliquots were thawed one by one and utilized in the animal trial. Yeast viability was confirmed by enumeration onto PDA plates.

Six-weeks-old CD1 male mice were purchased from Envigo Laboratories (Oderzo, Italy) and housed in groups of 2 per individually ventilated cage, in a temperature-controlled environment (22 ± 2°C) under a 12-h light-dark cycle. Chow food and water were provided *ad libitum*. Mice were allowed to acclimate to the laboratory for 1 week before entering experimentation. Nine groups of 10 mice were established, each group being assigned a yeast strain. The mice received for 14 days a daily dose of yeast biomass via gastric gavage. Controls were fed with 150 μL of PBS containing 15% v/v glycerol. At the end of the treatment and after 2, 4, and 8 weeks of washout, fecal pellets were collected from each mouse and immediately stored at -80°C in pre-weighted tubes containing 50% glycerol. Yeasts were counted onto PDA plates supplemented with 100 mg/L chloramphenicol and 100 mg/L ampicillin. Isolates were subjected to RAPD-PCR profiling, in order to check the identity with the supplemented strain. This study was carried out in compliance with the appropriate laws and institutional guidelines (DL n. 116/92 art. 5). The protocol was approved by the Animal Care and Use Ethics Committee of the University of Padua, in compliance with the national and European guidelines for handling and use of experimental animals.

### Statistics

In biofilm formation, adherence, and HBD-2 assays, the data are presented as means ± SD of at least three independent experiments. Means for *C. albicans*, NAC, and NCF groups were compared with one-way ANOVA followed by Bonferroni’s *post hoc* test and were considered significantly different for *P* < 0.05. In the colonization assay, the viable counts at 2, 4, and 8 weeks were compared to that at 0 weeks using Wilcoxon signed-rank test *T* and were considered significantly different for *P* < 0.05.

## Results

### Fungal Communities Based on Metagenomic ITS1 Sequencing

**FIGURE 1:**
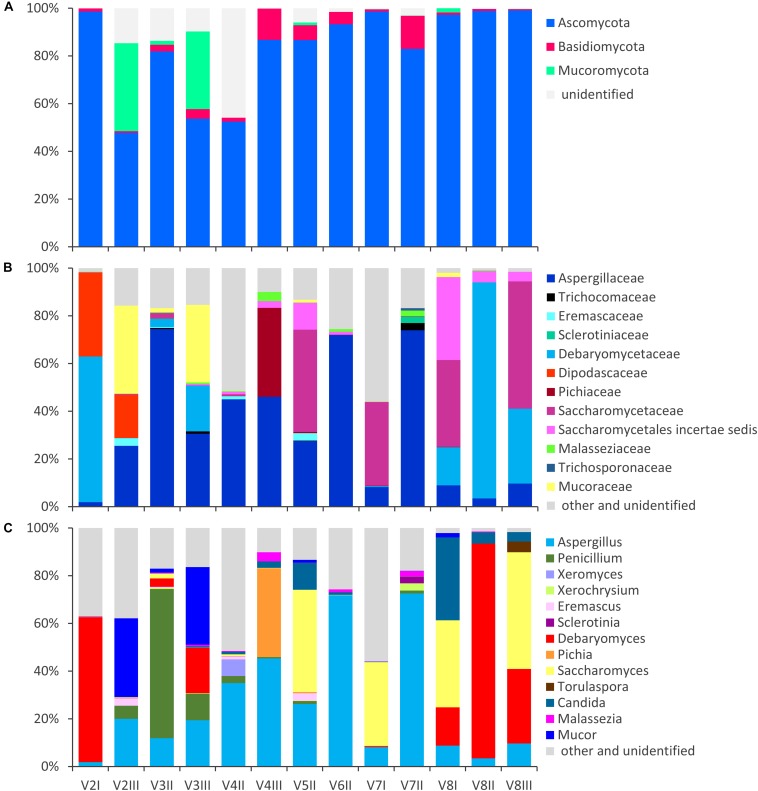
Stacked bar-plot representation of the relative abundances of the main fungal taxa in the intestinal mycobiota, based on metagenomic ITS1 sequences. Only the taxa with a taxonomic attribution at the level of phylum **(A)**, family **(B)**, and genus **(C)** and appearing at least once with abundance higher than 1% are reported, all the others being grouped as “other and unclassified.”

Fecal samples of eight healthy adults were collected at three time points across a period of 1 year, i.e., 0, 3, and 12 months, hereinafter referred to as samples I, II, and III, respectively, and subjected to the profiling fungal and bacterial communities using metagenomic ITS1 and 16S sequencing, respectively. Only 13 out of 24 samples provided a valuable number of fungal reads, that resulted in a total of 140,225 quality-trimmed ITS1 sequences (on average 10,787 reads per sample) and 103 OTUs net of singletons (on average 28.2 OTUs per sample, ranging from 11 to 40) (Figure 1 and Supplementary Datasheet S1). The unsuccessful sequencing was attributed to the too low concentration of fungal DNA, likely due to high concentrations of non-target DNA and to contaminants that may have been co-extracted with DNA. The 16S sequencing of the whole set of samples yielded 231,082 reads, attributed to 343 OTUs net of singletons (on average 130 OTUs per sample ranging between 81 and 178) (Supplementary Figure S1 and Supplementary Datasheet S2).

**FIGURE 2:**
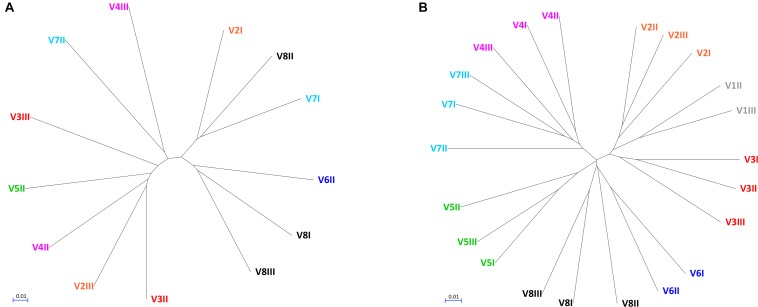
UPGMA cladograms of fungal **(A)** and bacterial **(B)** communities, constructed using the corresponding Jaccard distance matrices.

The alpha diversity indices (Chao1, Shannon, and Pielou), all lower for fungi compared to bacteria, indicated that fungal communities presented lower richness and evenness than the bacterial ones (Supplementary Figure S2). Fungal communities were also more dissimilar/distant from each other, while the bacterial ones presented evident subject-based groups with the Jaccard beta diversity metric (Figure 2). PERMANOVA analysis of beta diversity highlighted significant differences among bacterial communities from different subjects (*P* < 0.01), with the distance between the subjects being generally greater than that within each subject. With regards to the fungal community, no significant difference among subjects was observed (*P* > 0.05), resulting from great distances both among the subjects and in the same subject over the time.

The fungal OTUs were ascribed to Ascomycota (62), Basidiomycota (34), and Mucorimycota (5). Ascomycota and Basidiomycota were ubiquitously present across the samples, with the former, always being the most abundant, ranging from 47.8 to 99.5% and the latter from 0.5 to 13.9% (Figure 1). Mucoromycota were generally negligible (not detected or <2%) with the exception of the samples V2III and V3III (36.9 and 32.6%, respectively).

Within Ascomycota, the family Aspergillaceae was the most frequent and on average the most abundant. The genus *Aspergillus* was the most widespread and copious, accounting for up to 72.5% (mean = 25.7%), while *Penicillium* was less than 1% in most cases, albeit it dominated in sample V3II (62.6%). A number of *Aspergillus* and *Penicillium* species occurred sporadically and were negligible in terms of abundance, with the exception of *Aspergillus niger* that was highly recurrent (11/13), although being always less than 1%.

Other families of Ascomycota that reached remarkably high amounts in few samples were Debaryomycetaceae (mostly *Debaryomyces*), Dipodascaceae (unidentified genus), Saccharomycetaceae (mostly *Saccharomyces* and *Torulaspora*), Pichiaceae (*Pichia*), and the Saccharomycetales *incertae sedis* encompassing the genus *Candida*. *Debaryomyces udenii* and *S. cerevisiae* were the most frequent and abundant species. *D. udenii* was identified in 11 out of 13 samples, with relative amounts up to 89.5% (19.9% on average), while *S. cerevisiae* was detected in 12 of 13 samples, with relative amounts up to 48.9% (13.9% on average). High levels of *Pichia terricola* and *Torulaspora delbrueckii* characterized the samples V4III and V8III, respectively. The genus *Candida* occasionally occurred at remarkably high level, with only a few detected species (e.g., *C. ethanolica*, *C. inconspicua*, *C. parapsilosis*, *C. tropicalis*, and *C. zeylanoides*), that never accounted for more than 0.5%. *C. albicans* was never identified.

Basidiomycota mostly belonged to unclassified members of Tremellomycetes (up to 9.7%) and to family Malasseziaceae (genus *Malassezia*). *Malassezia restricta* was common (12/13), reaching up to 3.8% of abundance. *Mucor* encompassed most of the OTUs of Mucoromycota. *Mucor piriformis* and *Mucor circinelloides* were recurrent (8/13 and 7/13, respectively), reaching the remarkably high level of approx. 32% in some samples.

In order to establish whether a relationship could occur between bacterial and fungal genera, Spearman rank correlations between the genera were calculated on the basis of 16S and ITS1 metagenome analysis (data not shown). No significant correlations between bacterial and fungal genera were found, excluding the correlations resulting from bacterial and fungal genera occurring only in a sample.

### Enumeration, Isolation, and Taxonomic Identification of Cultivable Fungi

In parallel to NGS profiling, the fresh fecal samples were spread onto selective plates in order to determine the viable counts of fungi. In five samples, the fungal load was below the limit of detection (<10^2^ cfu/g), while it ranged from 1.4 × 10^2^ to 4.5 × 10^5^ cfu/g in the others (Table 1). A wide quantitative variability was detected over the time within a same subject, with a minority of cases presenting comparable fungal loads (*P* > 0.05) between different time points.

**TABLE 1:** Counts of cultivable fungi in the feces of eight healthy volunteers, collected at 0 (I), 3 (II), and 12 months (III).

**Subject**	**Cultivable fungi (cfu/g)**		
	**I**	**II**	**III**
V1	(8.1 ± 1.8) × 10^2*^	(9.9 ± 1.2) × 10^2*^	(3.6 ± 0.2) × 10^3^
V2	(2.9 ± 0.7) × 10^3^	(6.3 ± 0.8) × 10^2^	(4.5 ± 0.2) × 10^5^
V3	<10^2^	(2.6 ± 0.6) × 10^4*^	(2.1 ± 0.3) × 10^4*^
V4	(2.2 ± 0.9) × 10^3*^	(3.6 ± 0.6) × 10^2^	(1.4 ± 0.1) × 10^3*^
V5	(4.5 ± 1.7) × 10^2^	(3.3 ± 1.0) × 10^3^	(1.8 ± 0.2) × 10^3^
V6	(2.7 ± 0.6) × 10^2^	<10^2^	<10^2^
V7	<10^2^	<10^2^	(5.9 ± 0.6) × 10^2^
V8	(1.4 ± 0.1) × 10^4^	(4.7 ± 0.7) × 10^2^	(6.2 ± 0.3) × 10^4^

Values are means ± SD, *n* = 3. Within each row, for samples presenting detectable loads, ^*^ indicate means that did not significantly differ (*P* > 0.05).

Forty-eight colonies from each sample were randomly selected and clustered into a total of 27 biotypes based on their RAPD-PCR profile, referred to 11 genera and 17 species (Table 2). Some RAPD-PCR profiles of *C. albicans*, *C. parapsilosis*, *C. pararugosa*, *R. mucilaginosa*, and *I. terricola* were similar across different hosts, indicating that this technique was unsuitable for typing such isolates at the strain level. SSCP and MLP fingerprinting differentiated *C. albicans* isolates in 7 biotypes, each occurring in a single subject (Table 2).

**TABLE 2:** RAPD-PCR, SSCP, and MLP analysis of fungal isolates.

**Subject**	**I**			**II**			**III**		
	**Isolates**	Biotype^*^	**r.a. (%)**	**Isolates**	Biotype^*^	**r.a. (%)**	**Isolates**	Biotype^*^	**r.a. (%)**
V1	*Candida zeylanoides 01-03*	2	61	*Rhodotorula mucilaginosa 01-08*	13	38	*Candida zeylanoides 01-15*	2	47
	*Candida zeylanoides 01-04*	3	23	*Candida zeylanoides 01-11*	2	35	*Candida lusitaniae 01-17*	19	20
	*Candida pararugosa 01-07*	1	8	*Rhodotorula mucilaginosa 01-09*	10	9	*Rhodotorula mucilaginosa 01-20*	10	14
	*Exsofiala dermatitidis 01-06*	4	8	*Issatchenkia terricola 01-10*	11	9	*Candida parapsilosis 01-18*	9	7
				*Penicillium crustosum 01-12*	12	9	*Geotrichum candidum 01-19*	20	7
							*Pichia manshurica 01-14*	21	5
V2	*Geotrichum candidum 02-01*	5	86	*Geotrichum candidum 02-09*	14	86	*Geotrichum candidum 02-11*	14	100
	*Geotrichum candidum 02-02*	6	7	*Candida albicans 02-10*	8 A	14			
	*Geotrichum candidum 02-03*	7	7						
V3	–			*Saccharomyces cerevisiae 03-01*	15	100	*Debaryomyces hansenii 03-10*	23	58
							*Candida guilliermondii 03-03*	22	16
							*Candida parapsilosis 03-04*	9	12
							*Talaromyces purpureogenus 03-02*	25	7
							*Penicillium diversum 03-09*	24	5
							*Rhodotorula mucilaginosa 03-05*	10	2
V4	*Candida albicans 04-02*	8 B	88	*Aspergillus candidus 04-08*	16	50	*Candida albicans 04-10*	8 C	73
	*Rhodotorula mucilaginosa 04-01*	10	8	*Aspergillus candidus 04-21*	17	25	*Candida pararugosa 04-14*	1	23
	*Candida parapsilosis 04-04*	9	4	*Aspergillus candidus 04-22*	18	25	*Saccharomyces cerevisiae 04-11*	26	4
V5	*Candida albicans 05-01*	8 D	100	*Candida albicans 05-05*	8 D	84	*Candida albicans 05-09*	8 D	72
				*Rhodotorula mucilaginosa 05-06*	10	16	*Rhodotorula mucilaginosa 05-07*	10	28
V6	*Candida albicans 06-01*	8 E	100	–			–		
V7	–			–			*Candida albicans 07-02*	8 F	100
V8	*Candida albicans 08-06*	8 G	98	*Candida albicans 08-08*	8 G	98	*Saccharomyces cerevisiae 08-11*	27	100
	*Issatchenkia terricola 08-03*	11	2	*Rhodotorula mucilaginosa 08-07*	10	2			

Taxonomic characterization through ITS sequencing and relative abundance are reported for each cluster identified in the eight volunteers at 0 (I), 3 (II), and 12 months (III). – Indicates that no fungal colonies were isolated. ^*^Numbers indicate RAPD-PCR profiles; letters represent SSCP and MLP fingerprinting profiles of *Candida albicans* isolates. r.a., relative abundance expressed as %.

*Candida albicans* was never isolated in samples from V1 and V3, while it generally represented the sole or the dominant species among cultivable fungi in samples from the other six subjects. In subject V5, the *C. albicans* 8 D recurred at all the time points as the most abundant among cultivable fungi (72–100%). *C. zeylanoides* was found only in V1, with one of the two biotypes present at all the time-points as one of the most abundant fungal isolates (35–61%). The NAC isolates belonging to the species *C. parapsilosis*, *C. pararugosa*, *C. guillermondi*, and *C. lusitaniae* never occurred more than once in the same individual and accounted for 4–23% of the isolates.

*Geotrichum candidum* was isolated only from V1 and V2. In the latter subject, it presented five biotypes, one of which recurring across different time points and being always dominant among the isolates. *R. mucilaginosa* was isolated from five subjects. The species was particularly abundant in some samples (up to 47% of the isolates inV1-II). *S. cerevisiae*, *Debaryomyces hansenii*, and *Aspergillus candidus* were found only once in one or in a few subjects, but were generally abundant. *D. hansenii* was the dominant species in V3-III (58%).

### Biofilm Formation

**FIGURE 3:**
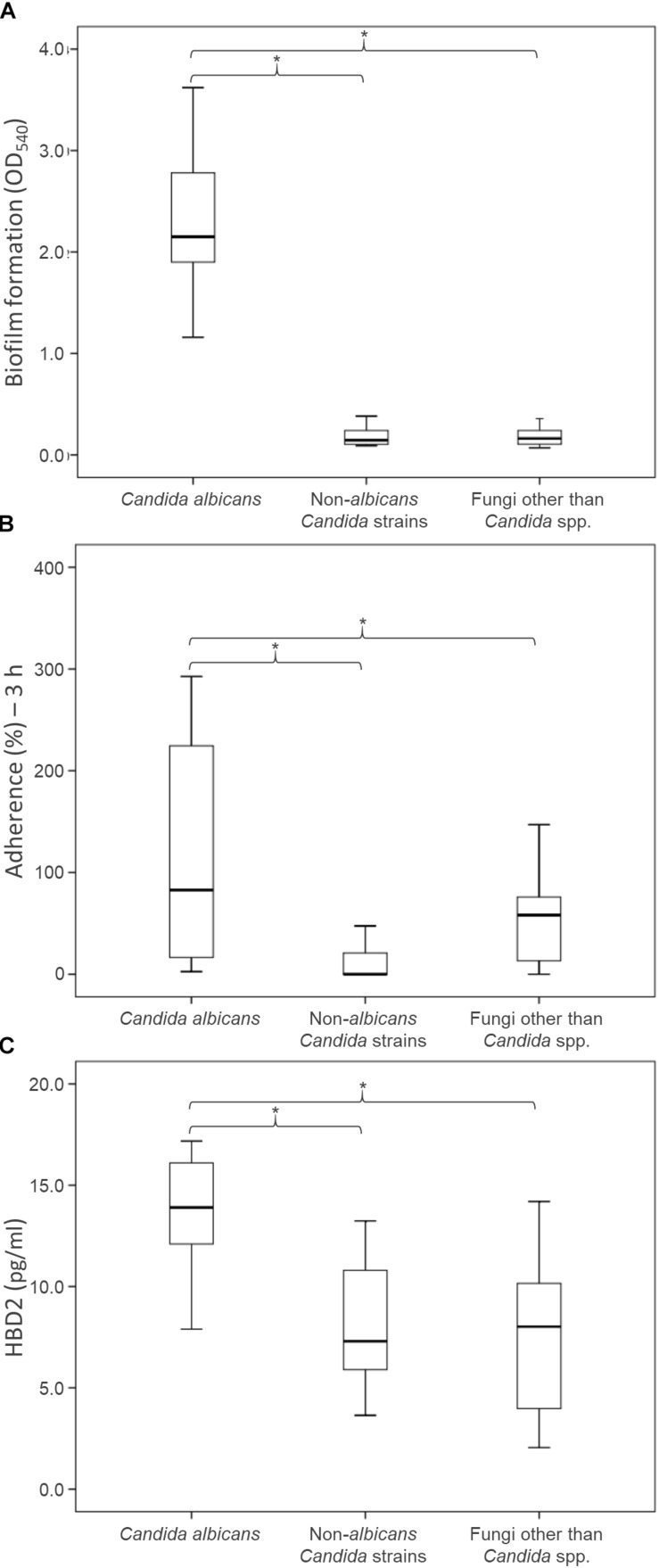
Health relevant properties of the fungi isolated from fecal specimens of healthy donors: biofilm production **(A)**, adhesion to epithelial Caco-2 cells **(B)**, and induction of HBD-2 production in CaCo-2 cells **(C)**. Boxes indicate the median and 25th and 75th percentiles; whiskers indicate 10th and 90th percentiles; ^*^indicate groups with significantly different means (*P* < 0.05).

The ability of the fungal isolates to form biofilm *in vitro* was investigated (Figure 3A; Supplementary Table S1). All but one of *C. albicans* isolates produced large amounts of biofilm after 48 h of incubation, yielding OD_540_ values higher than 1.5. Conversely, both the NAC and NCF did not form biofilm. On average, the OD_540_ was 2.39 ± 0.79 for *C. albicans*, 0.21 ± 0.13 for NAC, and 0.20 ± 0.14 for NCF. The ability of *C. albicans* to produce biofilms was significantly greater (*P* < 0.05) than that of NAC and NCF.

### Adherence of Fungal Isolates to Human Epithelial Cells Caco-2

An *in vitro* adhesion assay employing the human colorectal carcinoma cell line Caco-2 was used to evaluate the ability of the yeast isolates to adhere to intestinal epithelial cells (Figure 3B, Supplementary Table S1). The *C. albicans* isolates adhered more efficiently (*P* < 0.05) than NAC and NCF ones (13.4 ± 3.8, 8.1 ± 3.0, and 7.4 ± 3.9%, respectively). The percentage of adherence to Caco-2 cells was >11% for most *C. albicans*. Only two *C. albicans* isolates (i.e., 07-02 and 08-06) showed a quite low adherence capability. Conversely, the majority of NAC adhered less than 9%, with only few strains (*C. parapsilosis* 01-18, *C. pararugosa* 04-14, *C. zeylanoides* 01-03) showing values comparable to those of *C. albicans*. This feature was not species-specific, since other strains did not present similar behavior.

### Human β-Defensin 2 Production by Caco-2 Cells Exposed to Fungal Isolates

The secretion of HBD-2 by Caco-2 epithelial cells was analyzed after exposure to the fungal isolates (Figure 3C and Supplementary Table S1). The cells challenged with *C. albicans* produced higher amounts of HBD-2 than those treated with NAC or NCF (*P* < 0.05). The response of Caco-2 cells to the diverse isolates of *C. albicans* was quite different, with HBD-2 levels ranging between 2.6 and 592.7 pg/mL. In contrast, most of the NAC did not induce any HBD-2 production, with the exception of five strains that prompted the release of 2.0–47.5 pg/mL HBD-2. NCF caused a variable accumulation of HBD-2 (51.7 ± 40.6 pg/mL). *Issatchenkia terricola* and *Pichia manshurica* did not induce any HBD-2 production. *G. candidum* caused the highest HBD-2 release within NCF isolates, ranging between 71.4 and 110.9 pg/mL, followed by *R. mucilaginosa* (36.1–78.6 pg/mL), and by *S. cerevisiae* (8.5–18.5 pg/mL).

### Yeast Colonization of the Murine GIT

**FIGURE 4:**
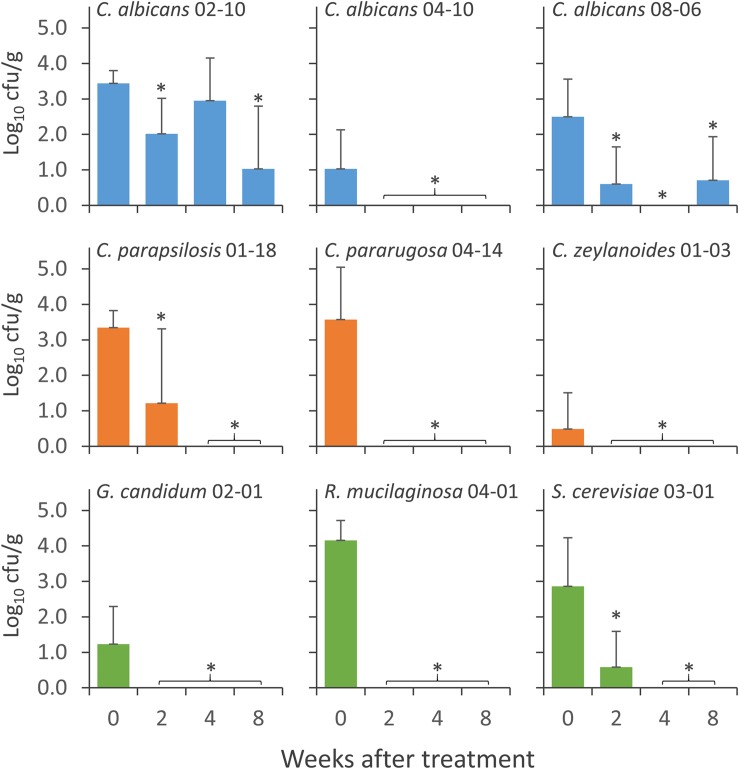
Concentration of the strains of *Candida albicans* (cyan), NAC (orange), and NCF (green) in the feces of mice groups during the wash period that followed the treatment with *C. albicans* 02-10, *C. albicans* 08-06, *C. albicans* 04-10, *C. parapsilosis* 01-18, *C. pararugosa* 04-14, *C. zeylanoides* 01-03, *G. candidum* 02-01 (G), *S. cerevisiae* 03-01 (H), and *R. mucilaginosa* 04-01 (I). Values are means (*n* = 10), the error bars representing the SD. For each strain, ^*^indicate statistically significant difference compared to 0 weeks values (Wilcoxon signed-rank test, *P* < 0.05).

Nine yeast isolates, i.e., 3 *C. albicans* (*C. albicans* 02-10, *C. albicans* 04-10, and *C. albicans* 08-06), 3 NAC (*C. parapsilosis* 01-18, *C. pararugosa* 04-14, and *C. zeylanoides* 01-03), and 3 NCF (*G. candidum* 02-01, *R. mucilaginosa* 04-01, and *S. cerevisiae* 03-01), were tested for the ability to colonize the GIT of mice. Yeasts were daily given to mice for 14 days and the persistence in feces was evaluated at the end of the treatment (0), then after 2, 4, and 8 weeks of washout (Figure 4). All the strains were recovered at the end of the supplementation period. The most abundant were *C. albicans* 02-10, *C. parapsilosis* 01-18, *C. pararugosa* 04-14, and *R. mucilaginosa* 04-01, all presenting a mean concentration >3.0 Log_10_ cfu/g. *C. albicans* 04-10 and *C. zeylanoides* 01-03 were the less abundant, with a mean concentration <1 Log_10_ cfu/g. *C. albicans* 04-10, the NAC, and the other yeasts dropped below the limit of detection after 2 or 4 weeks of washout. On the other hand, *C. albicans* 02-10 and *C. albicans* 08-06 were found for up to 8 weeks after the end of treatment. The former strain remained in the range of 1–3 Log_10_ cfu/g throughout the whole washout period, whereas the latter drastically decreased in the first 2 weeks, remaining present, although sporadically, in the feces of mice after 8 weeks.

## Discussion

This study investigated the fecal mycobiome in healthy adults over a time span of 12 months, using ITS1 deep sequencing combined with a culture-based method. The ITS profiling provided a comprehensive description of the fungi harbored in the intestine, whereas isolation of viable fungi allowed the study of the properties involved in gut colonization. Compared to the bacterial microbiome, the biodiversity of fungal communities was lower and characterized by greater unevenness, in agreement with previous studies (Qin et al., 2010; Suhr and Hallen-Adams, 2015; Nash et al., 2017). Most samples were dominated by one or two fungal genera. The unevenness in fungal composition resulted in great distances both among subjects and over the time within the same subject. This was consistent with the occurrence of fungal cells transiting through the GIT without being stable colonizers, or with the possibility of a high variability in the individual mycobiome. On the contrary, the bacterial microbiome yielded subject-based clusters coherent with a more stable resident community inhabiting the intestine of each donor.

Previous studies and the data herein presented consistently indicated *Aspergillus*, *Candida*, *Debaryomyces*, *Malassezia*, *Penicillium*, *Pichia*, and *Saccharomyces* as the most recurrent and/or dominant fungal genera. The major discrepancies with literature were associated with the detection of *Mucor* and to the absence or scarcity of some genera such as *Cladosporium*, *Clavispora*, *Cyberlindnera*, and *Galactomyces* (Hoffmann et al., 2013; Strati et al., 2016; Nash et al., 2017; Auchtung et al., 2018; Borges et al., 2018). Geographic distribution of the volunteers and diet habits probably accounted for the differences observed, since the present study and Strati’s investigation, both enrolling volunteers in Italy, come to similar conclusions. Many species of *Aspergillus* and *Penicillium* detected in this trial, each occurring sporadically and/or negligibly in terms of abundance, are regarded as health concerns, being potential sources of mycotoxins or opportunistic infections targeting mucosal tissues (Hedayati et al., 2007; Perrone and Gallo, 2017; Perrone and Susca, 2017). This supported the hypothesis of their transient presence in the gut as a result of the consumption of exogenous sources, such as contaminated food and beverages. In the GIT, fungal population can be made by true inhabitants or by microbes occasionally ingested with fermented or spoiled foods, likewise lactic acid bacteria abundantly occurring in food (Rossi et al., 2016). Despite *Aspergillus* and *Penicillium* were found in nearly all the samples, isolates ascribable to these genera were obtained only in a minority of cases. A similar observation was reported also by Strati et al. (2016) and suggests that fecal *Aspergillus* and *Penicillium* may be allochthons that do not survive the gastrointestinal transit. Likewise, other frequent and abundant taxa detected in the metagenomic survey (e.g., *Mucor*, *Malassezia*) were not identified by culture-based analysis, raising the question of whether they were actually viable inhabitants of the GIT.

The genus *Candida* was identified by metagenomics survey in most samples, occasionally occurring at remarkably high amounts. Unlike previous studies, *C. albicans* was not detected (Hoffmann et al., 2013; Seed, 2014; Suhr and Hallen-Adams, 2015; Strati et al., 2016; Suhr et al., 2016; Nash et al., 2017; Auchtung et al., 2018; Borges et al., 2018). Most of the *Candida* genus-related sequences received the “*Candida* unidentified” taxonomy using the last available version of the UNITE database (release 2017-12-01), but were given the designation “*Candida albicans*” if a previous version was utilized (release 2015-08-01) (Supplementary Figure S3). This underlines that the reliability of reference databases is pivotal for a consistent taxonomic attribution and for the comparison with other metagenomic studies and culture dependent approaches. As a matter of fact, isolates of *C. albicans* were frequently obtained and dominated the cultivable mycobiota of different samples. The molecular fingerprinting of the isolates lead to the identification of strains that longitudinally persisted within the same subject, likely as commensals inhabiting the intestine or systematically reaching it from the oral cavity.

Only few NAC species (*C. ethanolica*, *C. inconspicua*, *C. parapsilosis*, *C. tropicalis*, and *C. zeylanoides*) were detected by metagenome analysis. Evidence of a possible stable colonization was provided for *C. zeylanoides* by culture-dependent experiments, with a biotype isolated in the feces of a subject over a time span of one year. Based on the present metagenomics analysis, *S. cerevisiae* resulted as one of the most frequent and abundant species. Albeit cultivation of *S. cerevisiae* is generally not an issue, isolates were obtained only from two samples. This result was in agreement with a previous study, where the isolation succeeded only in a minority of samples (4/111) (Strati et al., 2016). Therefore, conclusive evidence was not obtained establishing whether *S. cerevisiae* is an occasional or a permanent colonizer of the human GIT. A recent study conducted a phylogenetic analysis on 12 microsatellite loci and 1715 combined CDSs from whole-genome sequencing on a set of *S. cerevisiae* strains isolated from the gut of normal and inflammatory bowel disease patients (Ramazzotti et al., 2019). The analysis revealed evidence of clonal colonization within the host’s gut. The phenotypic analysis of the isolates combined with immunological profiling indicated that both genetic and environmental factors involved in cell wall remodeling and sporulation are the main drivers of adaptation in *S. cerevisiae* populations in the human gut. The most likely hypothesis is that a combination of specific genetic features in the microbe and in the host allow colonization from microorganisms normally present solely as passengers, often when alterations in the gastrointestinal barrier occur (Sokol et al., 2017).

Fingerprinting of the NCF isolates pointed out that also the *G. candidum* and *R. mucilaginosa* may stably inhabit the GIT, even if genera *Geotrichum* and *Rhodotorula* were not detected by metagenome survey. However, the family of Dipodascaceae which includes the genus *Geotrichum* was found to be abundant in the corresponding samples. Interestingly, metagenomic revealed that also the ecologically understudied *D. udenii* is a putative permanent colonizer that, when detected, is present in all the samples of the same subject.

Functional investigation focused on comparison of *C. albicans*, NAC and NCF isolates. *C. albicans* adhered to human epithelial cells more efficiently than NAC and NCF and produced greater amounts of biofilm *in vitro*. In contrast, NAC and NCF were not biofilm producers. This evidence is in agreement with previous studies biofilm formation by of *C. albicans* and/or other fungi, such as *S. cerevisiae* (Chandra et al., 2001). Although the fungal population investigated in this study has been isolated from the stool of healthy volunteers, the ability to adhere and to form biofilm appears to be a trait peculiar of the opportunistic fungus *C. albicans*, the behavior of which supports the potential virulence and the impact on clinical infections.

Increasing evidence indicates that fungi may stimulate host intestinal immune system, beneficially affecting host homeostasis (Yan et al., 2013). The *C. albicans* isolates also induced the highest release of HBD-2 by human epithelial cells, further differing from NAC and NCF. On these bases, our results suggest that the release of HBD-2 by *C. albicans* may have an important role in regulating the size and the activity of the fungal population harbored in the gut of healthy individuals. The intestinal epithelial cells play a crucial role in local antimicrobial defense, since they not only represent a physiologic barrier for pathogens, but they also function as a part of innate immune system via production of antimicrobial peptides and cytokines, such as HBD-2, endowed with a wide spectrum of antibacterial and antifungal activity (Ganz, 2003; Selsted and Ouellette, 2005).

Three representative fungal isolates from each group have been administered to mice to evaluate the ability to colonize the intestine. Only two out of three *C. albicans* isolates were recovered from the stools of animals, 8 weeks after the end of the treatment. In contrast, the other tested fungi including NAC and NCF were not isolated yet after 2 weeks of wash-out. These results provide expand initial evidence on the allochthonous nature of most fungi in the gut, confirming that stability of mycobiota in the intestine over the time is quite low (Erturk-Hasdemir and Kasper, 2013).

## Conclusion

NGS approach combined with a culture-based methods revealed that most of the fungi transit through the GIT without being stable colonizers. The panel of fungi detected by metagenomic surveys is dominated by environmental or food-borne taxa that are rather washed out by the gastrointestinal transit, while only a minority of viable fungi behave as stable colonizers, with certain biotypes being longitudinally persistent within a subject. Notoriously, *C. albicans* exhibits features involved in stable colonization (e.g., transition to hyphal form, biofilm formation, adhesion to intestinal epithelial cells). Accordingly, it is the sole species that successfully colonized the murine intestine. It remains to be established if other species, herein identified as putative colonizers, such as *D. udenii*, *C. zeylanoides*, *G. candidum*, *S. cerevisiae*, and *R. mucilaginosa*, are true inhabitants of the intestine or are delocalized from other body districts. A combination of specific features in the microbe and in the host seems the driver of colonization of microorganisms normally present solely as passengers. As whole, the fungal microbiome is much more variable at the individual level than the bacterial one.

## Data Availability

The datasets generated for this study can be found in NCBI, PRJNA545913.

## Ethics Statement

The animal study was carried out in compliance with the appropriate laws and institutional guidelines (DL n. 116/92 art. 5). The protocol was approved by the Animal Care and Use Ethics Committee of the University of Padua, in compliance with the national and European guidelines for handling and use of experimental animals. For the collection of fecal samples from healthy human subject, written informed consent was obtained from all the enrolled subjects, in accordance with the protocol approved by the local research ethics committee (reference number 225-15, Comitato Etico Provinciale, Azienda Policlinico di Modena, Italy).

## Author Contributions

MR, DC, IC, SaP, and EB conceived and designed the experiments. AlA, FC, and DC performed the metagenomic study and the bioinformatics. SR, CG, MS, and LR isolated and characterized the cultivable fungi. AnA, SiP, and BC performed the phenotypical characterization of yeast isolates. PB and IC performed the animal trial. AlA and MR wrote the manuscript with contributions from all other authors.

## Conflict of Interest Statement

The authors declare that the research was conducted in the absence of any commercial or financial relationships that could be construed as a potential conflict of interest.

## Footnotes

1 https://unite.ut.ee/repository.php

2 https://www.arb-silva.de/download/arb-files/

## References

[B1] Albanese D. Fontana P. De Filippo C. Cavalieri D. Donati C. (2015). MICCA: a complete and accurate software for taxonomic profiling of metagenomic data. *Sci. Rep.* 5:9743. 10.1038/srep09743 25988396 PMC4649890

[B2] Auchtung T. A. Fofanova T. Y. Stewart C. J. Nash A. K. Wong M. C. Gesell J. R. (2018). Investigating colonization of the healthy adult gastrointestinal tract by fungi. *mSphere* 3:e00092-18. 10.1128/mSphere.00092-18 29600282 PMC5874442

[B3] Barelli C. Albanese D. Donati C. Pindo M. Dallago C. Rovero F. (2015). Habitat fragmentation is associated to gut microbiota diversity of an endangered primate: implications for conservation. *Sci. Rep.* 5:14862. 10.1038/srep14862 26445280 PMC4595646

[B4] Bolyen E. Rideout J. R. Dillon M. R. Bokulich N. A. Abnet C. Al-Ghalith G. A. (2018). QIIME 2: reproducible, interactive, scalable, and extensible microbiome data science. *PeerJ* 6:e27295v2. 10.1038/s41587-019-0209-9 PMC7015180 31341288

[B5] Borges F. M. de Paula T. O. Sarmiento M. R. A. de Oliveira M. G. Pereira M. L. M. Toledo I. V. (2018). Fungal diversity of human gut microbiota among eutrophic, overweight, and obese individuals based on aerobic culture-dependent approach. *Curr. Microbiol.* 75 726–735. 10.1007/s00284-018-1438-8 29368026

[B6] Brandt M. E. Lockhart S. R. (2012). Recent taxonomic developments with *Candida* and other opportunistic yeasts. *Curr. Fungal Infect. Rep.* 6 170–177. 10.1007/s12281-012-0094-x 26526658 PMC4626447

[B7] Cavalieri D. Di Paola M. Rizzetto L. Tocci N. De Filippo C. Lionetti P. (2018). Genomic and phenotypic variation in morphogenetic networks of two *Candida albicans* isolates subtends their different pathogenic potential. *Front. Immunol.* 8:1997. 10.3389/fimmu.2017.01997 29403478 PMC5780349

[B8] Chandra J. Kuhn D. M. Mukherjee P. K. Hoyer L. L. McCormick T. Ghannoum M. A. (2001). Biofilm formation by the fungal pathogen *Candida albicans*: development, architecture, and drug resistance. *J. Bacteriol.* 183 5385–5394. 10.1128/JB.183.18.5385-5394.2001 11514524 PMC95423

[B9] Erturk-Hasdemir D. Kasper D. L. (2013). Resident commensals shaping immunity. *Curr. Opin. Immunol.* 25 450–455. 10.1016/j.coi.2013.06.001 23830047 PMC3775925

[B10] Findley K. Oh J. Yang J. Conlan S. Deming C. Meyer J. A. (2013). Topographic diversity of fungal and bacterial communities in human skin. *Nature* 498 367–370. 10.1038/nature12171 23698366 PMC3711185

[B11] Ganz T. (2003). Defensins: antimicrobial peptides of innate immunity. *Nat. Rev. Immunol.* 3 710–720. 10.1038/nri1180 12949495

[B12] Hedayati M. T. Pasqualotto A. C. Warn P. A. Bowyer P. Denning D. W. (2007). *Aspergillus flavus*: human pathogen, allergen and mycotoxin producer. *Microbiology* 153 1677–1692. 10.1099/mic.0.2007/007641-0 17526826

[B13] Hoffmann C. Dollive S. Grunberg S. Chen J. Li H. Wu G. D. (2013). Archaea and fungi of the human gut microbiome: correlations with diet and bacterial residents. *PLoS One* 8:66019. 10.1371/journal.pone.0066019 23799070 PMC3684604

[B14] Huseyin C. E. O’Toole P. W. Cotter P. D. Scanlan P. D. (2017). Forgotten fungi-the gut mycobiome in human health and disease. *FEMS Microbiol. Rev.* 41 479–511. 10.1093/femsre/fuw047 28430946

[B15] Li J. Bai F. Y. (2007). Single-strand conformation polymorphism of microsatellite for rapid strain typing of *Candida albicans*. *Med. Mycol.* 45 629–635. 10.1080/13693780701530950 17885945

[B16] Mayer F. L. Wilson D. Hube B. (2013). *Candida albicans* pathogenicity mechanisms. *Virulence* 4 119–128. 10.4161/viru.22913 23302789 PMC3654610

[B17] Merseguel K. B. Nishikaku A. S. Rodrigues A. M. Padovan A. Ferreira R. C. de Azevedo Melo A. S. (2015). Genetic diversity of medically important and emerging *Candida* species causing invasive infection. *BMC Infect. Dis.* 15:57. 10.1186/s12879-015-0793-3 25887032 PMC4339437

[B18] Moyes D. L. Richardson J. P. Naglik J. R. (2015). *Candida albicans*-epithelial interactions and pathogenicity mechanisms: scratching the surface. *Virulence* 6 338–346. 10.1080/21505594.2015.1012981 25714110 PMC4601190

[B19] Muadcheingka T. Tantivitayakul P. (2015). Distribution of *Candida albicans* and non-*albicans Candida* species in oral candidiasis patients: correlation between cell surface hydrophobicity and biofilm forming activities. *Arch. Oral Biol.* 60 894–901. 10.1016/j.archoralbio.2015.03.002 25819801

[B20] Nash A. K. Auchtung T. A. Wong M. C. Smith D. P. Gesell J. R. Ross M. C. (2017). The gut mycobiome of the human microbiome project healthy cohort. *Microbiome* 5:153. 10.1186/s40168-017-0373-4 29178920 PMC5702186

[B21] Orsi C. F. Borghi E. Colombari B. Neglia R. G. Quaglino D. Ardizzoni A. (2014). Impact of *Candida albicans* hyphal wall protein 1 (HWP1) genotype on biofilm production and fungal susceptibility to microglial cells. *Microb. Pathog.* 69-70 20–27. 10.1016/j.micpath.2014.03.003 24685698

[B22] Perrone G. Gallo A. (2017). *Aspergillus* species and their associated mycotoxins. *Methods Mol. Biol.* 1542 33–49. 10.1007/978-1-4939-6707-0_3 27924530

[B23] Perrone G. Susca A. (2017). *Penicillium* species and their associated mycotoxins. *Methods Mol. Biol.* 1542 107–119. 10.1007/978-1-4939-6707-0_5 27924532

[B24] Qin J. Li R. Raes J. Arumugam M. Burgdorf K. S. Manichanh C. (2010). A human gut microbial gene catalogue established by metagenomic sequencing. *Nature* 464 59–65. 10.1038/nature08821 20203603 PMC3779803

[B25] Raimondi S. Amaretti A. Rossi M. Fall P. A. Tabanelli G. Gardini F. (2017). Evolution of microbial community and chemical properties of a sourdough during the production of Colomba, an Italian sweet leavened baked product. *LWT Food Sci. Technol.* 86 31–39. 10.1016/j.lwt.2017.07.042

[B26] Ramazzotti M. Stefanini I. Di Paola M. De Filippo C. Rizzetto L. Berná L. (2019). Population genomics reveals evolution and variation of *Saccharomyces cerevisiae* in the human and insects gut. *Environ. Microbiol.* 21 50–71. 10.1111/1462-2920.14422 30246283

[B27] Rossi M. Martínez-Martínez D. Amaretti A. Ulrici A. Raimondi S. Moya A. (2016). Mining metagenomic whole genome sequences revealed subdominant but constant Lactobacillus population in the human gut microbiota. *Environ. Microbiol. Rep.* 8 399–406. 10.1111/1758-2229.12405 27043715

[B28] Sampaio P. Gusmão L. Correia A. Alves C. Rodrigues A. G. Pina-Vaz C. (2005). New microsatellite multiplex PCR for *Candida albicans* strain typing reveals microevolutionary changes. *J. Clin. Microbiol.* 43 3869–3876. 10.1128/JCM.43.8.3869-3876.2005 16081924 PMC1233915

[B29] Seed P. C. (2014). The human mycobiome. *Cold Spring Harb. Perspect. Med.* 5:a019810. 10.1101/cshperspect.a019810 25384764 PMC4448585

[B30] Selsted M. E. Ouellette A. J. (2005). Mammalian defensins in the antimicrobial immune response. *Nat. Immunol.* 6 551–557. 10.1038/ni1206 15908936

[B31] Simon G. L. Gorbach S. L. (1984). Intestinal flora in health and disease. *Gastroenterology* 86 174–193. 10.1016/0016-5085(84)90606-1 6357937

[B32] Sokol H. Leducq V. Aschard H. Pham H. P. Jegou S. Landman C. (2017). Fungal microbiota dysbiosis in IBD. *Gut* 66 1039–1048. 10.1136/gutjnl-2015-310746 26843508 PMC5532459

[B33] Strati F. Di Paola M. Stefanini I. Albanese D. Rizzetto L. Lionetti P. (2016). Age and gender affect the composition of fungal population of the human gastrointestinal tract. *Front. Microbiol.* 7:1227. 10.3389/fmicb.2016.01227 27536299 PMC4971113

[B34] Suhr M. J. Banjara N. Hallen-Adams H. E. (2016). Sequence-based methods for detecting and evaluating the human gut mycobiome. *Lett. Appl. Microbiol.* 62 209–215. 10.1111/lam.12539 26669281

[B35] Suhr M. J. Hallen-Adams H. E. (2015). The human gut mycobiome: pitfalls and potentials–a mycologist’s perspective. *Mycologia* 107 1057–1073. 10.3852/15-147 26354806

[B36] Tang J. Iliev I. D. Brown J. Underhill D. M. Funari V. A. (2015). Mycobiome: approaches to analysis of intestinal fungi. *J. Immunol. Methods* 421 112–121. 10.1016/j.jim.2015.04.004 25891793 PMC4451377

[B37] Tsui C. Kong E. F. Jabra-Rizk M. A. (2016). Pathogenesis of *Candida albicans* biofilm. *Pathog. Dis.* 74: ftw018. 10.1093/femspd/ftw018 26960943 PMC5975230

[B38] Wang X. Liu C. Huang L. Bengtssonpalme J. Chen H. Zhang J. H. (2015). ITS1: a DNA barcode better than ITS2 in eukaryotes? *Mol. Ecol. Resour.* 15 573–586. 10.1111/1755-0998.12325 25187125

[B39] Wu P. F. Liu W. L. Hsieh M. H. Hii I. M. Lee Y. L. Lin Y. T. (2017). Epidemiology and antifungal susceptibility of candidemia isolates of non-albicans *Candida* species from cancer patients. *Emerg. Microbes Infect.* 6:e87. 10.1038/emi.2017.74 29018251 PMC5658770

[B40] Yan L. Yang C. Tang J. (2013). Disruption of the intestinal mucosal barrier in *Candida albicans* infections. *Microbiol. Res.* 168 389–395. 10.1016/j.micres.2013.02.008 23545353

[B41] Yang R. H. Su J. H. Shang J. J. Wu Y. Y. Li Y. Bao D. P. (2018). Evaluation of the ribosomal DNA internal transcribed spacer (ITS), specifically ITS1 and ITS2, for the analysis of fungal diversity by deep sequencing. *PLoS One* 13:e0206428. 10.1371/journal.pone.0206428 30359454 PMC6201957

