# Supplementary material for: Longitudinal Survey of Fungi in the Human Gut: ITS Profiling, Phenotyping, and Colonization
Source: Front Microbiol. 2019 Jul 10;10:1575. doi: 10.3389/fmicb.2019.01575 (PMC6636193; doi:10.3389/fmicb.2019.01575)
Supplement: Supplementary file 1 [file Table_1.docx]

Supplementary Material

Commensal and transient fungi of human gut:

from a metagenomic view to colonization studies

Stefano Raimondi, Alberto Amaretti, Caterina Gozzoli, Marta Simone, Lucia Righini, Francesco Candeliere, Paola Brun, Andrea Ardizzoni, Bruna Colomabari, Simona Paulone, Ignazio Castagliuolo, Duccio Cavalieri, Elisabetta Blasi, Maddalena Rossi*, and Samuele Peppoloni

* Correspondence: Maddalena Rossi, maddalena.rossi@unimore.it


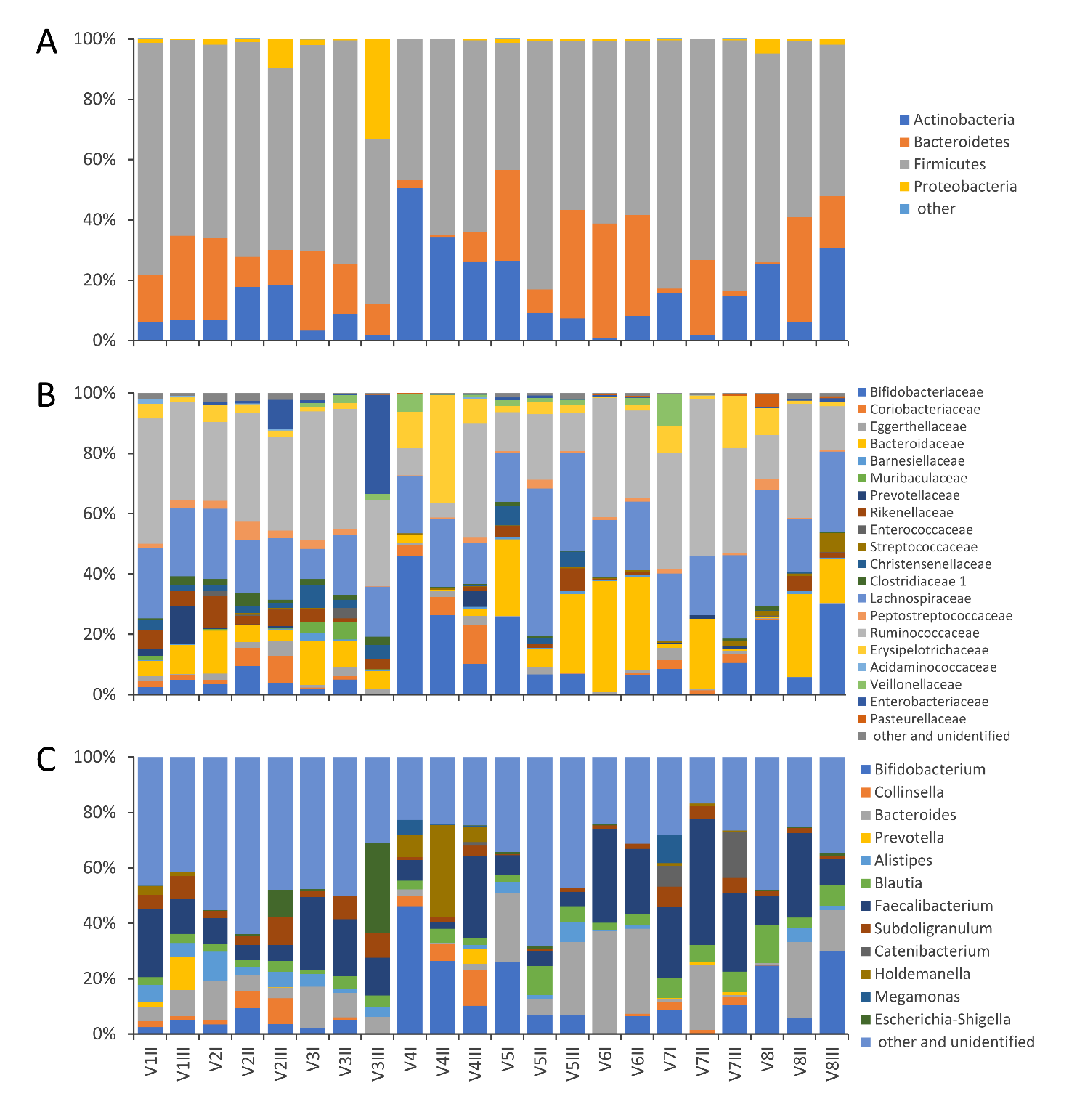
 **Supplementary figure S1**. Stacked bar-plot representation of the relative abundances of the main fungal taxa in the intestinal mycobiota, based on metagenomic pyrosequencing of 16S sequences. Only the taxa with a taxonomic attribution at the level of phylum (A), family (B), and genus (C) and appearing at least once with abundance higher than 5% are reported, all the others being grouped as ‘other and unclassified’.


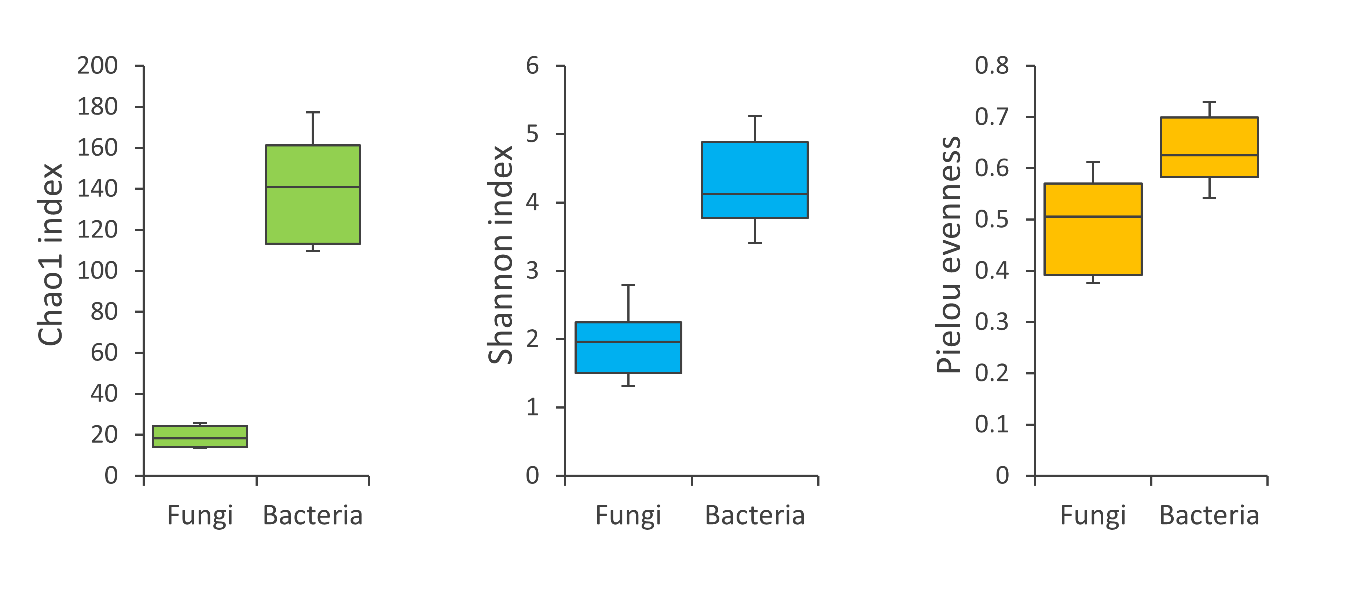


**Supplementary figure S2.** Alpha diversity indices (Chao1, Shannon, and Pielou) of the fungal and bacterial communities.

**Supplementary figure S3.** Comparison of the taxonomic attribution of *Candida* with the UNITE database 7.0 (release 2015-08-01) and 7.2 (release 2017-12-01).

**Supplementary Table 1.** Phenotypic characterization of fungal strains: biofilm formation *in vitro*, adherence to Caco-2 cells, and induction of HBD-2 production. Fungal isolates are grouped as C. albicans, non-*albicans* *Candida* (NAC), and non-*Candida* fungi (NCF). Stars indicate the strains selected for the animal trial. Values are means, n = 3, SD always < 10%.

|  | **Strain** | **Biofilm formation**  **OD_540_** | **Adherence**  **%** | **HBD-2**  **pg/ml** |
| --- | --- | --- | --- | --- |
| *C. albicans* | *C. albicans* SC5314 | 2.15 | 13.7 | 82.7 |
|  | *C. albicans* 02-10 * | 1.16 | 15.9 | 224.7 |
|  | *C. albicans* 04-02 | 1.96 | 13.8 | 6.0 |
|  | *C. albicans* 04-09 | 2.78 | 16.6 | 2.6 |
|  | *C. albicans* 04-10 * | 3.60 | 15.5 | 17.9 |
|  | *C. albicans* 05-01 | 1.73 | 13.9 | 121.0 |
|  | *C. albicans* 05-05 | 2.42 | 12.1 | 16.5 |
|  | *C. albicans* 05-09 | 3.56 | 17.2 | 8.5 |
|  | *C. albicans* 06-01 | 1.90 | 16.3 | 224.3 |
|  | *C. albicans* 07-02 | 1.73 | 4.3 | 40.9 |
|  | *C. albicans* 08-01 | 2.44 | 16.1 | 592.7 |
|  | *C. albicans* 08-06 * | 2.08 | 7.9 | 292.7 |
|  | *C. albicans* 08-08 | 3.62 | 11.1 | 224.5 |
|  | **Mean ± SD** | **2.39 ± 0.79** | **13.4 ± 3.8** | **142.7 ± 170.1** |
| NAC | *C. guillermondii* 03-03 | 0.48 | 8.0 | 6.2 |
|  | *C. lusitaniae* 01-17 | 0.32 | 5.6 | 27.4 |
|  | *C. parapsilosis* 01-18 * | 0.22 | 12.5 | 0 |
|  | *C. parapsilosis* 03-04 | 0.17 | 3.6 | 0 |
|  | *C. parapsilosis* 04-04 | 0.12 | 8.5 | 0 |
|  | *C. pararugosa* 01-07 | 0.24 | 5.9 | 0 |
|  | *C. pararugosa* 04-14 * | 0.38 | 13.2 | 20.9 |
|  | *C. zeylanoides* 01-03 * | 0.09 | 10.8 | 2.0 |
|  | *C. zeylanoides* 01-04 | 0.09 | 7.1 | 0 |
|  | *C. zeylanoides* 01-11 | 0.11 | 7.5 | 47.5 |
|  | *C. zeylanoides* 01-15 | 0.10 | 6.7 | 0 |
|  | **Mean ± SD** | **0.21 ± 0.13** | **8.1 ± 3.0** | **9.5 ± 15.8** |
| NCF | *D. hansenii* 03-10 | 0.09 | 6.0 | 15.6 |
|  | *E. dermatitidis* 01-06 | 0.52 | 4.0 | 21.8 |
|  | *G. candidum* 01-19 | 0.12 | 2.1 | 81.4 |
|  | *G. candidum* 02-09 | 0.19 | 2.5 | 147 |
|  | *G. candidum* 02-11 | 0.13 | 3.3 | 83.9 |
|  | *G. candidum* 02-01 * | 0.09 | 8.5 | 110.9 |
|  | *G. candidum* 02-02 | 0.07 | 14.2 | 71.4 |
|  | *G. candidum* 02-08 | 0.16 | 5.2 | 98.5 |
|  | *I. terricola* 01-10 | 0.11 | 3.9 | 0 |
|  | *I. terricola* 08-03 | 0.07 | 10.5 | 0 |
|  | *P. manshurica* 01-14 | 0.36 | 4.4 | 0 |
|  | *R. mucilaginosa* 01-08 | 0.48 | 8.3 | 58.2 |
|  | *R. mucilaginosa* 01-09 | 0.10 | 8.2 | 71.5 |
|  | *R. mucilaginosa* 01-20 | 0.18 | 11.4 | 58.9 |
|  | *R. mucilaginosa* 03-05 | 0.20 | 6.2 | 27.7 |
|  | *R. mucilaginosa* 04-01 * | 0.08 | 3.2 | 65.2 |
|  | *R. mucilaginosa* 05-06 | 0.26 | 10.0 | 78.6 |
|  | *R. mucilaginosa* 05-07 | 0.22 | 8.8 | 36.1 |
|  | *R. mucilaginosa* 08-07 | 0.18 | 10.3 | 73.0 |
|  | *S. cerevisiae* 03-01 * | 0.46 | 3.4 | 18.5 |
|  | *S. cerevisiae* 04-11 | 0.16 | 14.2 | 10.9 |
|  | *S. cerevisiae* 08-11 | 0.11 | 13.9 | 8.5 |
|  | **Mean ± SD** | **0.20 ± 0.14** | **7.4 ± 3.9** | **51.7 ± 40.6** |
